# The Intersection of Socioeconomic Differences and Sex in the Management and Outcomes of Acute Myocardial Infarction: A Nationwide Cohort Study

**DOI:** 10.1177/00033197241273433

**Published:** 2024-09-19

**Authors:** Nicholas Weight, Saadiq Moledina, Claire A. Lawson, Harriette G. C. Van Spall, Harindra C. Wijeysundera, Muhammad Rashid, Evangelos Kontopantelis, Mamas A. Mamas

**Affiliations:** 1 Keele Cardiovascular Research Group, Centre for Prognosis Research, Institute for Primary Care and Health Sciences, 4212 Keele University, Keele, UK; 2 Department of Cardiovascular Sciences, 4488 University of Leicester, Leicester, UK; 3 Department of Medicine and Department of Health Research Methods, Evidence, and Impact, 3710 McMaster University, Hamilton, ON, Canada; 4 Population Health Research Institute, Research Institute of St Joe’s, Hamilton, ON, Canada; 5 206712 Institute of Health Policy, Management and Evaluation, University of Toronto, Toronto, ON, Canada; 6 Institute for Clinical Evaluative Sciences, Toronto, Canada; 7 Schulich Heart Program, Sunnybrook Health Sciences Centre, 5292 University of Toronto, Toronto, ON, Canada; 8 Temerty Faculty of Medicine, University of Toronto, Toronto, Canada; 9 Department of Cardiovascular Sciences, Glenfield Hospital, University Hospitals of Leicester NHS Trust Leicester United Kingdom; 10 NIHR Leicester Biomedical Research Centre, University of Leicester, Leicester, UK; 11 Division of Informatics, Imaging and Data Sciences, University of Manchester, Manchester, UK; 12 National Institute for Health and Care Research (NIHR) Birmingham Biomedical Research Centre, UK

**Keywords:** deprivation, acute myocardial infarction, angiography, percutaneous coronary intervention

## Abstract

Patients with lower socioeconomic status (SES) have poorer outcomes following acute myocardial infarction (AMI) than patients with higher SES; however, how sex modifies socioeconomic differences is unclear. Using the United Kingdom (UK) Myocardial Ischaemia National Audit Project (MINAP) registry, alongside Office of National Statistics (ONS) mortality data, we analyzed 736,420 AMI patients between 2005 and 2018, stratified by Index of Multiple Deprivation (IMD) score Quintiles (most affluent [Q1] to most deprived [Q5]). There was no significant difference in probability of in-hospital mortality in our adjusted model according to sex. The probability of 30-day mortality in our adjusted model was similar between men and women throughout Quintiles, ((Q5; Men 7.6%; 95% CI 7.3–7.8% (*P* < .001), Women; 7.0%; 95% CI 6.8–7.3%, *P* < .001)) ((Q1; Men 7.1%; 95% CI 6.8–7.4%, *P* < .001, Women; 6.9%; 95% CI 6.6–7.1%, *P* < .001)). The probability of one-year mortality in our adjusted model was higher in men throughout all Quintiles (Q1; Men 15.0%; 95% CI 14.8–15.6%), *P* < .001, Women; 14.5%; 95% CI 14.2–14.9%, *P* < .001) (Q5; Men 16.9%; 95% CI 16.5–17.3%, *P* < .001, Women; 15.5%; 95% CI 15.1–15.9 by %, *P* < .001). Overall, female sex did not significantly influence the effect of deprivation on AMI processes of care and outcomes.

## Introduction

Cardiovascular disease is the leading cause of mortality in women worldwide.^1^ Despite this, women still receive a poorer quality of care and have worse clinical outcomes compared with men following acute myocardial infarction (AMI). This includes being less likely to undergo invasive coronary angiography (ICA), revascularization by percutaneous coronary intervention (PCI) or coronary artery bypass graft (CABG) surgery, and having poorer in-hospital and longer-term clinical outcomes.^2-4^

Although the United Kingdom (UK) has a publicly funded, universal healthcare system, there are still documented disparities in access to care according to socioeconomic status (SES).^5^ The UK uses the Index of Multiple Deprivation (IMD), a comprehensive measure of deprivation including 38 separate indicators,^6,7^ to produce reports of the geographical spread of deprivation in the UK. Compared with the most affluent patients, those from the most deprived areas have been reported to have longer length of stay and a higher incidence of major adverse cardiovascular events (MACE) post-PCI,^8^ and by using the Welsh IMD score equivalent, it has been shown that patients from the most deprived quintile have higher 30-day mortality from AMI than those from the most affluent quintile.^9^

Despite the known sex and SES differences in cardiovascular care and outcomes, very few studies have explored the inter-relationships between sex and socioeconomic status. One study pointed to poorer outcomes following AMI in the most deprived regions, being more pronounced in women,^10^ and another that efforts to address inequities in processes of care for the patients from the most deprived areas have been less effective in women.^11^ But there is very limited data reporting on the interaction between sex and SES on the longer-term outcomes of AMI.

Thus, our study used the MINAP registry, in combination with Office of National Statistics (ONS) mortality data to assess the intersection of SES and sex in the processes of care and in-hospital, 30-day and 1-year mortality of AMI in the UK.

## Methods

### Study Design

We used MINAP, a prospective national registry of patients admitted to hospitals in the UK, with an acute coronary syndrome (ACS).^12^ The MINAP dataset consists of 130 variables, including baseline demographics and clinical characteristics, comorbidities, management strategies, pharmacotherapy, in-hospital clinical outcomes, and discharge diagnosis.^13^ Data are submitted by hospital clinical and clerical staff, and approximately 90,000 pseudonymized records annually are uploaded to the National Institute for Cardiovascular Outcomes Research (NICOR). In-hospital mortality is recorded in MINAP, but for longer-term mortality analysis, we used data from the Office of National Statistics (ONS).

### Study Population

We included patients admitted with a diagnosis of AMI in any of the 230 participating hospitals in England and Wales between January 2005 and March 2019. The discharge diagnosis of AMI was determined by local clinicians according to presenting history, clinical examination, and the results of in-patient investigations in keeping with the consensus document of the Joint European Society of Cardiology (ESC) and American College of Cardiology (ACC).^14^ Patients were excluded if they had missing data in our key variables for investigation; IMD score, in-hospital mortality, and major adverse cardiovascular events (MACE).

### Exposures

This constituted a final cohort of 736,420 patients with AMI, who were then divided into five quintiles, according to their IMD 2010 score. This is a comprehensive measure of deprivation including 38 separate indicators.^6^ Quintile 1 is the most (the most affluent group) in and Quintile 5 (the most deprived group) includes the highest 20% of IMD scores). IMD is widely used within the UK to classify the relative deprivation of different geographical areas.^6^ Multiple components of deprivation are weighted and compiled into a single score of deprivation. The IMD 2010 score comprised seven major domains: income deprivation, employment deprivation, health deprivation, disability, education, skills and training, barriers to housing and services, crime, and living environment. Domain scores are calculated from multiple relevant indicators, and these are then weighted into the overall IMD score, where pre-defined score cut-offs exist to create Quintiles.^6^

### Outcomes

#### Primary

Primary outcomes of interest included in-hospital all-cause mortality, 30-day mortality, and 1-year mortality. Thirty-days and 1-year mortality was calculated from date of admission with AMI (as recorded in MINAP) and date of death as recorded by the ONS.

#### Secondary

Secondary outcomes of interest included MACE (composite endpoint of in-patient all-cause mortality and reinfarction), cardiac mortality (death attributable to myocardial ischaemia or infarction, heart failure (HF), and cardiac arrest of unknown cause), and major bleeding.

### Statistical Analysis

Demographics, clinical characteristics, and crude adverse outcomes of patients by quintile of deprivation were compared using Pearson’s chi-square test for categorical variables. Continuous variables were compared using Students *t*-test, if normally distributed, and using the Wilcoxon Rank Sum test if not. Normality was assessed using the Shapiro–Wilk test. Continuous variables are presented as medians and interquartile ranges (IQR) and categorical variables as frequencies and proportions. Multiple imputations with chained equations (MICE) was used to impute values for variables with missing data, with all covariates and outcomes used in the analytical models included in the imputation process. Multiple imputations with chained equation is considered to be the best practice when dealing with situations where data is missing at random and can provide unbiased estimates even when levels of missing data are significant, and also some protection when the pattern of ‘missingness’ is not random.^15^ Multivariable logistic regression analysis was applied to imputed datasets, for each binary outcome of interest, to estimate the risk of adverse outcomes between quintiles of deprivation and sex. Estimates were combined using Rubin’s rules.^16^ Logistic regression models were fitted using maximum likelihood estimation and were adjusted for age, sex, year ethnicity, heart rate, blood pressure, history of AMI, co-morbid conditions (family history of coronary artery disease, hypertension, hypercholesterolaemia, diabetes, chronic renal failure, smoking, history of asthma or chronic obstructive pulmonary disease (COPD) history of cerebrovascular accident (CVA) or peripheral arterial disease (PAD), pharmacotherapy (prescription of low molecular weight heparin (LMWH), warfarin, aspirin, ACE inhibitor, statins, beta-blockers and P2Y12 inhibitor), cardiac arrest, and procedures including invasive coronary angiography (ICA) and revascularisation (by percutaneous coronary intervention (PCI) or coronary artery bypass graft surgery (CABG) during admission). Analyses were performed to compare the patients’ baseline characteristics, management strategies, and outcomes in the five quintiles according to gender. For descriptive tables, we formed two further groups according to gender as recorded in the MINAP dataset; Male and Female. We conducted our analysis of the marginal effect of IMD Quintile and gender using each sex as captured in MINAP. The margins command on Stata 14.2 was used to quantify the independent association of combinations of IMD Quintiles and sex classification according to the MINAP data dictionary, on adjusted in-hospital mortality, 30 -day mortality and 1-year mortality. These are probabilities for each group, which were then plotted using the ‘marginsplot’ function. Statistical analysis was undertaken using Stata 14.2 (College Station, Texas, USA). All statistical analyses were two-tailed, with an alpha of 5% used.

## Results

### Baseline Clinical Characteristics according to Quintile of Deprivation

**Figure 1.**
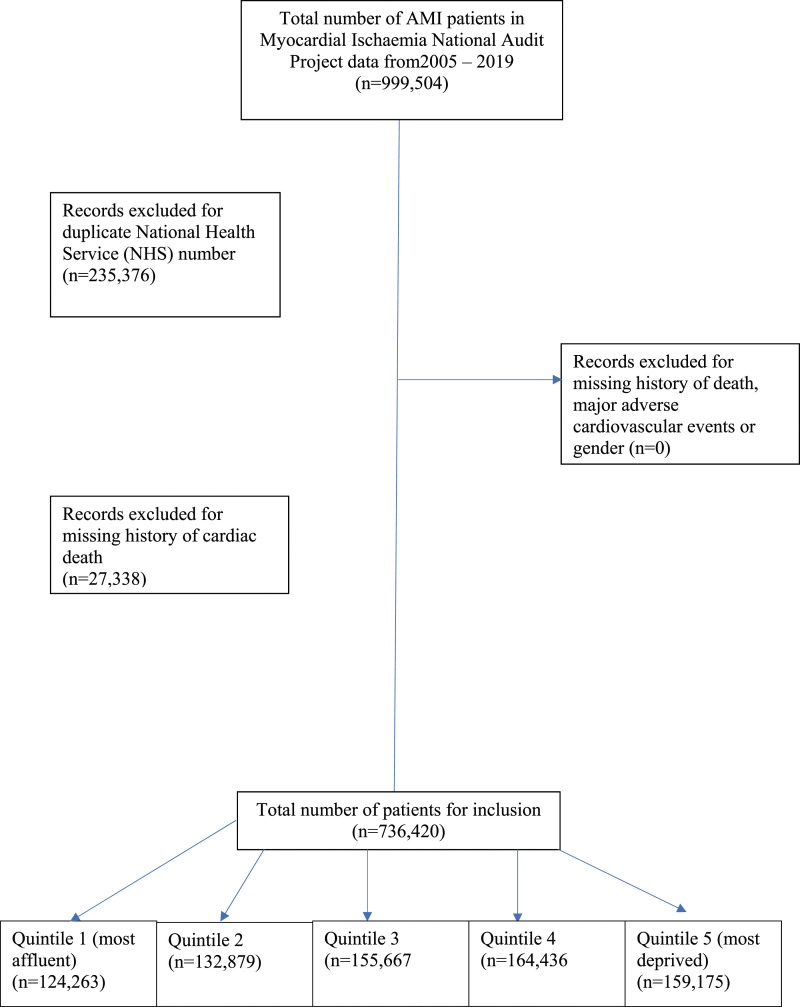
STROBE diagram detailing exclusion criteria.

After applying the exclusion criteria, we had a study population of 736,420 AMI patients from the UK between January 2005 and August 2019, which were split into five quintiles of deprivation according to their 2010 IMD score as recorded in the MINAP registry (Figure 1). Patients from the most deprived Quintile (Quintile 5) had a younger median age (in years) (Quintile 5 (most deprived); 67 (56–78) versus Quintile 1 (most affluent);73 (62–82) years, had a higher body mass index (BMI) (Quintile 5; 27.5 (24.2–31.4) versus Quintile 1; 26.6 (23.8–29.8)) and were more likely to be from an ethnic-minority background (Quintile 5; 13% vs Quintile 1; 3%) (Table 1). Compared with the most affluent group, the most deprived group were more likely to be current smokers (Quintile 5; 40% vs Quintile 1; 18%), (*P* < .001) have diabetes mellitus (DM) (Quintile 5; 26% vs Quintile 1; 18%) (*P* < .001), and more often had a previous history of myocardial infarction (MI) (Quintile 5; 28% vs Quintile 1; 23%) (*P* < .001).

**Table 1.** Demographic Comparison Between IMD Score Quintiles for Patients Suffering AMI.

Variables	Quintile 1 (Most Affluent) (n = 124,263	Quintile 2 (n = 132,879	Quintile 3 (n = 155,667)	Quintile 4 (n = 164,436	Quintile 5 (Most Deprived) (n = 159,175	*P*
Age, years, median (IQR)	73 (62-82)	72 (61-81)	71 (60-81)	70 (58-80)	67 (56-78)	
Women (%)	40,442/124,263 (33)	43,858/132,879 (33)	52,385/155,667 (34)	56,791/164,436 (35)	56,159/159,175 (35)	<.001
BMI, median [IQR]	26.5 (23.8–29.7)	26.7 (24.0–30.1)	26.9 (24.0–30.8)	27.1 (24.0–30.8)	27.4 (24.1–31.2)	
Ethnicity – White	60,152/62,315 (97)	63,564/66,397 (96)	73,214/78,132 (94)	74,122/82,358 (90)	70,679/80,517 (88)	<.001
Ethnicity – Asian	1,866/62,315 (3)	2,417/66,397 (4)	4,105/78,132 (5)	6,684/82,358 (8)	8,260/80,517 (10)	<.001
Ethnicity – Black	202/62,315 (0)	299/66,397 (0)	640/78,132 (1)	1,296/82,358 (2)	1,336/80,517 (2)	<.001
Ethnicity – Mixed	95/62,315 (0)	117/66,397 (0)	173/78,132 (0)	256/82,358 (0)	242/80,517 (0)	<.001
Basal crepitations (%)	6,797/57,395 (12)	7,572/60,695 (12)	9,299/71,101 (13)	10,212/76,100 (13)	9,178/70,423 (13)	<.001
Pulmonary oedema (%)	2,743/57,395 (5)	3,017/60,695 (5)	3,469/71,101 (5)	3,845/76,110 (5)	3,801/70,423 (5)	<.001
Cardiogenic shock (%)	1,031/57,395	1,102/60,695 (2)	1,374/71,101 (2)	1,553/76,110 (2)	1,385/70,423 (2)	<.001
High risk GRACE score >140 (%)	37,372/54,722 (68)	38,749/57,971 (67)	44,788/67,861 (66)	45,965/72,628 (63)	40,377/67,840 (60)	<.001
Intermediate risk GRACE score 109–140 (%)	13,050/54,722 (24)	14,330/57,971 (25)	16,938/67,861 (25)	19,133/72,628 (26)	19,072/67,840 (28)	<.001
Low risk GRACE score <109 (%)	4,300/54,722 (8)	4,892/57,971 (8)	6,135/67,861 (9)	7,530/72,628 (10)	8,391/67,840 (12)	<.001
ECG ST changes (%)	104,163/119,84 (87)	111,643/128,488 (87)	130,434/150,931 (86)	137,737/159,300 (86)	133,446/153,557 (87)	<.001
Previous smoker (%)	41,009/114,863 (36)	43,963/123,407 (36)	50,045/144,152 (35)	49,709/152,712 (33)	43,649/148,692 (29)	<.001
Current smoker (%)	20,328/114,863 (18)	26,771/123,407 (22)	36,451/144,152 (25)	48,089/152,712 (31)	59,853/148,692 (40)	<.001
Chronic renal failure (%)	6,455/115,245 (6)	7,026/121,421 (6)	8,299/141,585 (6)	8,975/148,954 (6)	8,403/140,543 (6)	<.001
Prior percutaneous coronary intervention (%)	8,381/116,047 (7)	9,014/121,994 (7)	10,588/142,559 (7)	11,135/149,387 (7)	10,297/140,878 (7)	<.001
Diabetes (%)	20,712/120,827 (17)	24,194/128,992 (19)	30,761/150,831 (20)	36,358/159,172 (23)	37,323/153,308 (24)	<.001
CCF (%)	5,694/115,367 (5)	6,254/121,564 (5)	7,703/141,885 (5)	8,279/149,244 (6)	8,009/140,726 (6)	<.001
Hypercholesterolaemia (%)	35,833/114,533 (31)	37,783/121,154 (31)	44,045/141,729 (31)	48,442/148,621 (33)	46,556/140,436 (33)	<.001
Previous MI (%)	18,773/116,960 (16)	20,896/123,889 (17)	25,051/145,330 (17)	27,750/152,270 (18)	27,675/142,812 (19)	<.001
History of angina (%)	24,300/116,097 (21)	26,691/122,771 (22)	31,990/143,572 (22)	34,993/150,448 (23)	34,862/141,744 (25)	<.001
Cerebrovascular disease (%)	9,022/115,211 (8)	9,625/121,588 (8)	11,751/141,996 (8)	12,768/149,357 (9)	12,493/140,842 (9)	<.001
Peripheral artery disease (%)	4,324/113,952 (4)	4,851/120,387 (4)	5,949/140,891 (4)	6,604/148,102 (4)	7,103/139,741 (5)	<.001
Hypertension (%)	58,681/117,149 (50)	61,691/123,831 (50)	72,576/144,972 (50)	76,728/151,978 (50)	70,394/143,372 (49)	<.001
Asthma/COPD (%)	13,695/114,222 (12)	16,186/120,778 (13)	20,399/141,295 (14)	24,527/148,695 (16)	27,659/140,464 (20)	<.001
Family history of CAD (%)	28,965/95,891 (30)	30,780/101,275 (30)	34,663/116,941 (30)	37,638/123,340 (31)	38,343/116,503 (33)	<.001
Heart rate, bpm, median (IQR)	76 (65-90)	77 (65-90)	78 (66-92)	79 (66-92)	80 (67-94)	
Systolic blood pressure, median (IQR)	138 (120-157)	138 (129-157)	137 (120-156)	137 (119-156)	137 (118-156)	
Serum creatinine (micromol/L)	89 (74–109)	89 (74–111)	88 (74–109)	88 (73–109)	87 (72–108)	
Good LV function (%)	31,757/88,991 (36)	34,257/94,740 (36)	38,990/108,951 (35)	42,217/115,384 (37)	39,254/108,942 (36)	<.001
Moderate LVSD (%)	19,512/88,991 (22)	21,043/94,740 (22)	24,073/108,951 (22)	25,847/115,384 (22)	24,634/108,942 (23)	<.001
Severe LVSD (%)	96,147/88,991 (7)	6,685/94,740 (7)	8,063/108,951 (7)	8,764/115,384 (8)	8,629/108,942	<.001
Cardiac arrest (%)	8,550/121,122 (7)	8,804/129,640 (7)	10,316/151,915 (7)	11,017/159,841 (7)	10,257/152,582 (7)	<.001
Previous CABG surgery (%)	6,806/116,281 (6)	7,163/122,242 (6)	8,289/142,823 (6)	8,449/149,662 (6)	7,433/141,128 (5)	<.001
Admission to coronary care unit (%)	67,881/123,425 (55)	72,628/131,892 (55)	83,792/154,381 (54)	89,374/162,843 (55)	85,835/157,618 (54)	<.001
Admission to cardiology ward (%)	12,297/123,425 (10)	13,591/131,892 (10)	14,974/154,381 (10)	15,019/162,843 (9)	16,718/157,618 (11)	<.001
Admission under consultant cardiologist (%)	71,339/122,145 (58)	75,793/130,430 (58)	86,565/152,441 (57)	92,939/160,856 (58)	93,017/154,203 (60)	<.001

*Abbreviations*: CABG; coronary artery bypass graft, LVSD; left ventricular systolic dysfunction, CAD; coronary artery disease, COPD; chronic obstructive pulmonary disease, MI; myocardial infarction, CCF; congestive cardiac failure, BMI; body mass index, GRACE; global registry of acute coronary events, IQR; interquartile range.

### Management and Clinical Outcomes according to Quintile of Deprivation

Patients in the most deprived group were statistically less likely to undergo invasive coronary angiography (ICA) (Quintile 5; 67% vs Quintile 1; 69% (*P* < .001)) or uring their index admission although differences were small (Table 2). In our unadjusted analyses, there was a small difference in the likelihood of in-hospital mortality (Quintile 5; 6% vs Quintile 1; 7% (*P* < .001)) and MACE (Quintile 5; 7% vs Quintile 1; 8% (*P* < .001)) between the most deprived and most affluent quintiles.

**Table 2.** Management Strategy and Clinical Outcome Comparison Between IMD 2010 Quintiles for Patients Suffering from AMI.

Variables	Quintile 1 (Most Affluent) (n = 124,263	Quintile 2 (n = 132,879	Quintile 3 (n = 155,667)	Quintile 4 (n = 164,436	Quintile 5 (Most Deprived) (n = 159,175	*P*
Low molecular weight heparin (%)	61,190/107,787 (57)	62,653/112,910 (55)	71,696/130,163 (55)	74,240/137,337 (54)	64,705/123,752 (52)	<.001
Fondaparinux (%)	27,833/93,084 (30)	29,263/96,861 (30)	34,085/111,301 (31)	35,556/117,350 (30)	32,763/104,324 (31)	<.001
Warfarin (%)	5,934/106,364 (6)	5,974/111,036 (5)	6,960/127,477 (5)	6,924/135,223 (5)	6,127/122,110 (5)	<.001
Unfractionated heparin (%)	30,259/106,517 (28)	29,872/110,985 (27)	33,565/127,359 (26)	34,837/135,236 (26)	30,249/122,179 (25)	<.001
Glycoprotein 2b/3a inhibitor (%)	9,895/108,081 (9)	9,748/113,149 (9)	11,275/131,027 (9)	12,371/137,936 (9)	11,061/124,047 (9)	<.001
IV nitrate (%)	19,339/106,478 (18)	19,938/111,094 (18)	22,884/127,535 (18)	23,842/135,353 (18)	20,087/122,103 (16)	<.001
IV beta-blockers (%)	2,538/107,200 (2)	2,420/112,137 (2)	2,682/129,381 (2)	2,942/136,534 (2)	2,630/122,690 (2)	<.001
MRA (%)	5,898/78,742 (7)	6,228/81,628 (8)	7,032/93,415 (8)	7,397/98,473 (8)	6,643/86,973 (8)	<.001
Aspirin (%)	93,513/97,037 (96)	100,486/104,053 (97)	116,799/121,087 (96)	123,550/128,113 (96)	120,418/124,330 (97)	<.001
P2Y12 inhibitor (%)	104,466/120,024 (87)	110,795/127,744 (87)	128,745/149,395 (86)	135,339/158,407 (85)	127,979/151,336 (85)	<.001
Statins (%)	101,469/120,579 (84)	109,178/128,984 (85)	127,395/150,664 (85)	135,482/159,132 (85)	132,686/152,934 (87)	<.001
ACE inhibitors/ARB (%)	92,622/121,735 (76)	98,974/129,957 (76)	115,006/152,164 (76)	121,752/160,697 (76)	118,480/154,270 (77)	<.001
Beta-blockers (%)	9,106/121,348 (74)	96,115/129,535 (74)	111,557/151,598 (74)	117,534/160,158 (73)	113,153/153,424 (74)	<.001
Cardiac rehabilitation (%)	92,294/113,429 (81)	98,044/120,588 (81)	112,921/140,564 (80)	118,365/148,416 (80)	117,636/143,135 (82)	<.001
Coronary angiogram (%)	83,985/121,343 (69)	88,589/129,307 (69)	102,813/151,290 (68)	107,777/159,150 (68)	103,377/153,249 (67)	<.001
Percutaneous coronary intervention (%)	54,241/122,123 (44)	57,364/130,393 (44)	66,320/152,739 (43)	71,092/161,168 (44)	68,685/156,266 (44)	<.001
CABG surgery (%)	2,504/97,676 (3)	2,818/103,555 (3)	3,283/120,531 (3)	3,690/126,811 (3)	3,467/119,804 (3)	<.001
Revascularization (CABG surgery/PCI) (%)	56,630/122,123 (46)	60,056/130,393 (46)	69,473/152,739 (45)	74,631/161,168 (46)	72,009/156,266 (46)	<.001
In-hospital mortality (%)	8,678/124,263 (7)	8,866/132,879 (7)	10,526/155,667 (7)	11,311/164,436 (7)	10,220/159,175 (6)	<.001
Thirty-day mortality (%)	10,140/124,263 (8)	10,570/132,879 (8)	12,453/155,667) (8)	13,332/164,436 (8)	12,145/159,175 (8)	<.001
One-year mortality (%)	20,646/124,263 (17)	21,969/132,879 (17)	26,049/155,667 (17)	28,099/164,436 (17)	26,162/159,175 (16)	<.001
Cardiac mortality (%)	7,093/124,263 (6)	7,439/132,879 (6)	8,751/155,667 (6)	9,370/164,436 (6)	8,494/159,175 (5)	<.001
Reinfarct (%)	2,825/179,168 (1)	1,804/120,745 (1)	2,088/141,496 (1)	2,101/148,509 (1)	1,918/141,009 (1)	.005
Major bleeding (%)	2,088/120,432 (2)	2,285/128,228 (2)	2,506/150,135 (2)	2,817/158,655 (2)	2,493/152,623 (2)	<.001
MACE (%)	9,904/124,263 (8)	10,284/132,879 (8)	12,120/155,667 (8)	12,898/164,436 (8)	11,723/159,175 (7)	<.001

*Abbreviations*: IV; intravenous, MRA; mineralocorticoid receptor antagonist, ACE: angiotensin-converting-enzyme, ARB; angiotensin receptor blockers, CABG; coronary artery bypass graft, PCI; percutaneous coronary intervention, MACE; major adverse cardiovascular events.

### Adjusted Clinical Outcomes according to Quintile of Deprivation

After adjusting for age, sex, year ethnicity, heart rate, blood pressure, history of AMI, co-morbid conditions (family history of coronary artery disease, hypertension, hypercholesterolaemia, diabetes, chronic renal failure, smoking, history of asthma or chronic obstructive pulmonary disease (COPD) history of cerebrovascular accident (CVA) or peripheral arterial disease (PAD), pharmacotherapy (prescription of low molecular weight heparin (LMWH), warfarin, aspirin, ACE inhibitor, statins, beta-blockers, and P2Y12 inhibitor), cardiac arrest and procedures including invasive coronary angiography (ICA), and revascularisation (by percutaneous coronary intervention (PCI) or coronary artery bypass graft surgery (CABG) during admission), there were significant differences in in-hospital mortality, MACE, 30-day mortality, and cardiac mortality when comparing the most deprived quintile with the most affluent quintile as a reference group (Table 3). One-year mortality after the same adjustment was more likely in the most deprived patient group (OR: 1.18, 95% CI; 1.14–1.23, *P* < .001).

**Table 3.** Adjusted Outcomes for Each Quintile of Deprivation Compared to the Most Affluent Quintile (Quintile 1).

Outcome Variables	Quintile 2 (n = 199,841)		Quintile 3 (n = 199,669)		Quintile 4 (n = 199,952)		Quintile 5 (Most Deprived) n = 200,631)	
	OR	*P*	OR	*P*	OR	*P*	OR	*P*
Primary outcomes								
MACE (in-hospital)	1.00 (.94–1.06)	.952	1.00 (.94–1.07)	.921	1.04 (.98–1.10)	.183	1.08 (1.01–1.15)	.026
In-hospital mortality	.97 (.90–1.05)	.486	1.00 (.93–1.08)	.927	1.06 (.99–1.14)	.097	1.09 (1.01–1.19)	.034
Thirty-day mortality	1.01 (.95–1.07)	.736	1.02 (.97–1.08)	.406	1.05 (1.00–1.11)	.071	1.10 (1.03–1.11)	.03
One-year mortality	1.03 (.99–1.06)	.133	1.06 (1.03–1.10)	<.001	1.13 (1.09–1.17)	<.001	1.18 (1.14–1.23)	<.001

Each quintile of deprivation is compared against our reference group ‘Quintile 1’, which is our most affluent quintile according to IMD score. Adjusted for: age, sex, year, ethnicity, heart rate, blood pressure, history of AMI, co-morbid conditions (family history of coronary artery disease, hypertension, hypercholesterolemia, diabetes, chronic renal failure, smoking, history of asthma or chronic obstructive pulmonary disease (COPD) history of cerebrovascular accident (CVA) or peripheral arterial disease (PAD), pharmacotherapy (prescription of low molecular weight heparin (LMWH), warfarin, aspirin, ACE inhibitor, statins, beta-blockers and P2Y12 inhibitor), cardiac arrest and procedures including invasive coronary angiography (ICA) and revascularization (by percutaneous coronary intervention (PCI) or coronary artery bypass graft surgery (CABG) during admission).

### Baseline Clinical Characteristics according to Sex and Quintile of Deprivation

Women had a significantly higher median age (in years) than men in all quintiles of deprivation (Quintile 5; Men 63.8, Women 73.1 vs Quintile 1; Men 69.7, Women 78.8). Women were less likely to be from an ethnic minority background (Quintile 5; Men 14%, Women 10% vs Quintile 1; Men 4%, Women 3%) (Table 4). Women were significantly less frequently current smokers (Quintile 5; Men 44%, Women 34% vs Quintile 1; Men 20%, Women 13%). Women more frequently had a previous diagnosis of hypertension (Quintile 5; Men 46%, Women 55% vs Quintile 1; Men 47%, Women 57%).

**Table 4.** Demographic Comparison Between IMD Score Quintiles Stratified by Sex.

Variables	Quintile 1 (Most Affluent) (n = 124,263		Quintile 2 (n = 132,879		Quintile 3 (n = 155,667)		Quintile 4 (n = 164,436		Quintile 5 (Most Deprived) (n = 159,175	
Gender	Male (n = 83,821)	Female (n = 40,442)	Male (n = 89,021)	Female (n = 43,858)	Male (n = 103,282)	Female (n = 52,385)	Male (n = 107,645)	Female (n = 56,791)	Male (n = 103,016)	Female (n = 56,159)
Age, years, median (IQR)	69.7 (59.5 -79.2)	78.8 (69.7-85.8)	68.8 (58.9 -78.5)	78.2 (68.4-85.3)	68.1 (57.9 78.0)	77.3 (67.1-84.9)	66.1 (55.9 -76.6)	75.9 (64.8-84.1)	63.8 (53.8- 74.7)	73.1 (61.4-82.1)
Ethnicity – White (%)	40,663/42,283 (96)	19,489/20,032 (97)	42,583/44,720 (95)	20,981/21,677 (97)	48,597/52,247 (93)	24,617/25,885 (95)	48,244/54,244 (89)	25,878/28,114 (92)	45,077/52,216 (86)	25,602/28,301 (90)
Ethnicity – Asian (%)	1,417/42,283 (3)	449/20,032 (2)	1,845/44,720 (4)	572/21,677 (3)	3,088/52,247 (6)	1,017/25,885	4,983/54,244 (9)	1,701/28,114 (6)	6,101/52,216 (12)	2,159/28,301 (8)
Ethnicity – Black (%)	139/42,283 (0)	63/20,032 (0)	204/44,720 (0)	95/21,677 (0)	434/52,247 (2)	206/25,885 (1)	838/54,244 (2)	458/28,114 (2)	861/52,216 (2)	475/28,301 (2)
Ethnicity – Mixed (%)	64/42,283 (0)	31/20,032 (0)	88/44,720 (0)	29/21,677 (0)	128/52,247 (0)	45/25,885 (0)	179/54,244 (0)	77/28,114 (0)	177/52,216 (0)	65/28,301 (0)
BMI, median [IQR]	26.9 (24.4–30.0)	25.5 (22.4–29.3)	27.1 (24.5–30.2)	26.0 (22.7–29.9)	27.3 (24.5–30.6)	26.1 (22.8–30.3)	27.4 (24.5–30.9)	26.6 (23.0–31.0)	27.5 (24.4–31.1)	27.3 (23.3–31.7)
Basal crepitations (%)	3,999/39,141 (10)	2,798/18,254 (15)	4,439/41,144 (11)	3,133/19,551 (16)	5,562/47,910 (12)	3,737/23,191 (16)	5,921/50,539 (12)	4,291/25,571 (17)	5,281/46,043 (11)	3,897/24,380 (16)
Pulmonary oedema (%)	1,621/39,141 (4)	1,122/18,254 (6)	1,794/41,144 (4)	1,223/19,551 (6)	2,044/47,910 (4)	1,425/23,191 (6)	2,146/50,539 (4)	1,699/25,571 (7)	2,166/46,043 (5)	1,635/24,380 (7)
Cardiogenic shock (%)	729/39,141 (2)	302/18,254 (2)	746/41,144 (2)	356/19,551 (2)	927/47,910 (2)	447/23,191 (2)	1,082/50,539 (2)	471/25,571 (2)	872/46,043 (2)	513/24,380 (2)
High risk GRACE score >140 (%)	23,935/37,272 (64)	13,437/17,450 (77)	24,520/39,251 (62)	14,229/18,720 (76)	28,291/45,670 (62)	16,497/22,191 (74)	28,179/48,125 (59)	17,786/24,503 (73)	24,367/44,284 (55)	16,010/23,556 (68)
Intermediate risk GRACE score 109–140 (%)	9,890/37,272 (27)	3,160/17,450 (18)	10,842/39,251 (28)	3,488/18,720 (19)	12,623/45,670 (28)	4,315/22,191 (19)	14,187/48,125 (29)	4,946/24,503 (20)	13,675/44,284 (30)	5,397/23,556 (23)
Low risk GRACE score <109 (%)	3,447/37,272 (9)	853/17,450 (5)	3,889/39,251 (10)	1,003/18,720 (5)	4,756/45,670 (10)	1,379/22,191 (6)	5,759/48,125 (12)	1,771/24,503 (7)	6,242/44,284 (14)	2,149/23,556 (9)
ECG ST changes (%)	70,365/80,886 (87)	33,798/38,961 (87)	74,799/86,157 (87)	36,844/42,331 (87)	86,518/100,236 (86)	43,916/50,695 (87)	90,306/104,414 (86)	47,431/54,886 (86)	86,470/99,484 (87)	46,976/54,073 (87)
Previous smoker (%)	31,122/78,097 (40)	9,887/36,766 (27)	33,103/83,263 (40)	10,860/40,144 (27)	33,103/83,263 (40)	10,860/40,144 (27)	36,019/100,823 (36)	13,690/51,889 (26)	30,426/96,729 (31)	13,223/51,963 (25)
Current smoker (%)	15,433/78,097 (20)	4,895/36,766 (13)	20,034/83,263 (24)	6,737/40,144 (17)	20,034/83,263 (24)	6,737/40,144 (17)	35,031/100,823 (35)	13,058/51,889 (25)	42,360/96,729 (44)	17,493/51,963 (34)
Chronic renal failure (%)	4,151/77,637 (5)	2,304/37,608 (6)	4,440/81,241 (5)	2,586/40,180 (6)	5,213/93,718) (6)	3,086/47,867 (6)	5,466/97,251 (6)	3,509/51,703 (7)	4,957/90,676 (5)	3,446/49,867 (7)
Prior percutaneous coronary intervention (%)	6,496/78,282 (8)	1,885/37,765 (5)	6,881/81,691 (8)	2,126/40,303 (5)	8,048/94,496 (9)	2,540/48,063 (5)	8,380/97,580 (9)	2,755/51,807 (5)	7,501/90,937 (8)	2,796/49,941 (6)
Diabetes (%)	14,021/81,489 (17)	6,691/39,338 (17)	16,135/86,366 (19)	8,059/42,626 (19)	20,355/100,032 (20)	10,406/50,799 (20)	23,306/104,083 (22)	13,052/55,089 (24)	23,302/99,105 (24)	14,021/54,203 (26)
CCF (%)	3,304/77,765 (4)	2,390/37,602 (6)	3,613/81,317 (4)	2,641/40,247 (7)	4,496/93,932 (5)	3,207/47,953 (7)	4,606/97,446 (5)	3,673/51,798 (7)	4,461/90,805 (5)	3,548/49,921 (7)
Hypercholesterolaemia (%)	24,944/77,240 (32)	10,889/37,293 (29)	24,944/77,240 (32)	11,780/40,013 (29)	30,017/93,928 (32)	14,028/47,801 (29)	32,393/97,119 (33)	16,049/51,502 (31)	30,305/90,694 (33)	16,251/49,742 (33)
History of angina (%)	16,475/78,279 (21)	7,825/37,818 (21)	17,749/82,159 (22)	8,942/40,612 (22)	20,925/95,118 (22)	11,065/48,454 (23)	22,540/98,248 (23)	12,453/52,200 (24)	21,787/91,464 (24)	13,075/50,280 (26)
Previous MI (%)	13,426/78,859 (17)	5,347/38,101 (14)	14,881/82,929 (18)	6,015/40,960 (15)	17,503/96,309 (18)	7,548/49,021 (15)	19,212/99,454 (19)	8,538/52,816 (16)	18,893/92,172 (20)	8,782/50,640 (17)
Cerebrovascular disease (%)	5,348/77,625 (7)	3,674/37,586 (10)	5,721/81,353 (7)	3,904/40,235 (10)	6,964/94,010 (7)	4,787/47,986 (10)	7,532/97,538 (8)	5,236/51,819 (10)	7,294/90,890 (8)	5,199/49,952 (10)
Peripheral artery disease (%)	3,031/76,771 (4)	1,293/37,181 (3)	3,392/80,553 (4)	1,459/39,834 (4)	4,096/93,302 (4)	1,853/47,589 (4)	4,605/96,730 (5)	1,999/51,372 (4)	4,898/90,200 (5)	2,205/49,541 (4)
Hypertension (%)	37,035/78,917 (47)	21,646/38,232 (57)	38,734/82,864 (47)	22,957/40,967 (56)	45,129/95,980 (47)	27,447/48,992 (56)	46,994/99,192 (47)	29,734/52,786 (56)	42,368/92,423 (46)	28,026/50,949 (55)
Asthma/COPD (%)	8,304/76,913 (11)	5,391/37,309 (14)	9,807/80,827 (12)	6,379/39,951 (16)	12,180/93,513 (13)	8,219/47,782 (17)	14,155/97,006 (15)	10,372/51,689 (20)	15,398/90,578 (17)	12,261/49,886 (25)
Family history of CAD (%)	20,985/65,566 (32)	7,980/30,325 (26)	21,968/68,726 (32)	8,812/32,549 (27)	24,443/78,535 (31)	10,220/38,406 (27)	26,189/81,710 (32)	11,449/41,630 (28)	26,006/76,146 (34)	12,337/40,357 (31)
Heart rate, bpm, median (IQR)	75 (64–88)	80 (68–94)	75 (64–89)	80 (68–95)	76 (65–90)	80 (68–95)	77 (65–90)	81 (69–96)	78 (66–91)	82 (69–97)
Systolic blood pressure, median (IQR)	137 (120–155)	140 (120–160)	137 (120–155)	139 (120–160)	137 (119–155))	139 (120–159)	136 (119–155)	138 (118–159)	136 (118–155)	138 (118–158)
Creatinine (micromol/L)	93 (79–111)	79 (65–101)	92 (79–113)	79 (65–102)	92 (78–113)	79 (65–102)	91 (77–111)	79 (65–103)	90 (76–110)	79 (64–102)
Good LV function (%)	21,732/60,184 (36)	10,025/28,807 (35)	23,325/63,701 (37)	10,932/31,039 (35)	26,256/72,618 (36)	12,734/36,333 (35)	27,968/76,029 (37)	14,249/39,355 (36)	25,555/71,020 (36)	13,699/37,922 (36)
Moderate LVSD (%)	13,584/60,184 (23)	5,928/28,807 (21)	14,628/63,701 (23)	6,415/31,039 (21)	16,730/72,618 (23)	7,343/36,333 (20)	17,845/76,029 (23)	8,002/39,355 (20)	16,782/71,020 (24)	7,852/37,922 (21)
Severe LVSD (%)	4,316/60,184 (7)	1,831/28,807 (6)	4,713/63,701 (7)	1,972/31,039 (6)	5,636/72,618 (8)	2,427/36,333 (7)	6,003/76,029 (8)	2,761/39,355 (7)	5,927/71,020 (8)	2,702/37,922 (7)
Cardiac arrest (%)	6,001/81,651 (7)	2,549/39,471 (6)	6,104/86,825 (7)	2,700/42,815 (6)	7,151/100,810 (7)	3,165/51,105 (6)	7,399/104,604 (7)	3,618/55,237 (7)	6,954/98,868 (7)	3,303/53,714 (6)
Previous CABG surgery (%)	5,534/78,440 (7)	1,272/37,841 (3)	5,840/81,869 (7)	1,323/40,373 (3)	6,611/94,676 (7)	1,678/48,147 (3)	6,715/97,760 (7)	1,734/51,902 (3)	5,676/91,125 (6)	1,757/50,003 (4)
Admission to coronary care unit (%)	48,653/83,249 (58)	19,228/40,176 (48)	51,508/88,352 (58)	21,120/43,540 (49)	59,179/102,410 (58)	24,613/51,971 (47)	62,060/106,594 (58)	27,314/56,249 (49)	58,879/102,008 (58)	26,956/55,610 (48)
Admission to cardiology ward (%)	8,414/83,249 (10)	3,883/40,176 (10)	9,212/88,352 (10)	4,379/43,540 (10)	10,070/102,410 (10)	4,904/51,971 (9)	10,053/106,594 (9)	4,966/56,249 (9)	10,929/102,008 (11)	5,789/55,610 (10)
Admission under consultant cardiologist (%)	51,097/82,406 (62)	20,242/39,739 (51)	53,634/87,409 (61)	22,159/43,021 (52)	61,021/101,136 (60)	25,544/51,305 (50)	64,784/105,344 (61)	28,155/55,512 (51)	63,450/99,860 (64)	29,567/54,343 (54)

*Abbreviations:* CABG; coronary artery bypass graft, LVSD; left ventricular systolic dysfunction, CAD; coronary artery disease, COPD; chronic obstructive pulmonary disease, MI; myocardial infarction, CCF; congestive cardiac failure, BMI; body mass index, GRACE; global registry of acute coronary events, IQR; interquartile range.

### Management and Clinical Outcomes according to Sex and Quintile of Deprivation

Women were less often treated with a P2Y12 inhibitor (Quintile 5; Men 85%, Women 83% vs Quintile 1; Men 88%, Women 85% (*P* < .001)) (Table 5). Women were significantly less likely to undergo ICA (Quintile 5; Men 72%, Women 58% vs Quintile 1; Men 75%, Women 57% (*P* < .001) and PCI (Quintile 5; Men 49%, Women 36% vs Quintile 1; Men 50%, Women 34% (*P* < .001)) during their admission when compared with men.

**Table 5.** Management Strategy and Clinical Outcome Comparison Between IMD Quintiles Stratified by Sex.

Variables	Quintile 1 (Most Affluent) (n = 124,263		Quintile 2 (n = 132,879		Quintile 3 (n = 155,667)		Quintile 4 (n = 164,436		Quintile 5 (Most Deprived) (n = 159,175	
Gender	Male (n = 83,821)	Female (n = 40,442)	Male (n = 89,021)	Female (n = 43,858)	Male (n = 103,282)	Female (n = 52,385)	Male (n = 107,645)	Female (n = 56,791)	Male (n = 103,016)	Female (n = 56,159)
Low molecular weight heparin (%)	40,504/72,547 (56)	20,686/35,240 (59)	41,187/75,404 (55)	21,466/37,506 (57)	46,588/86,061 (54)	25,108/44,102 (57)	47,436/89,432 (53)	26,804/47,905 (56)	41,021/79,592 (52)	23,684/44,160 (54)
Fondaparinux (%)	18,100/62,601 (29)	9,733/30,483 (32)	19,038/64,686 (29)	10,225/32,175 (32)	21,976/73,642 (30)	12,109/37,659 (32)	22,657/76,722 (30)	12,899/40,628 (32)	20,454/67,310 (30)	12,309/37,014 (33)
Warfarin (%)	3,820/71,528 (5)	2,114/34,836 (6)	3,903/74,073 (5)	2,071/36,963 (6)	4,458/84,112 (5)	2,502/43,365 (6)	4,325/88,012 (5)	2,599/47,211 (6)	3,786/78,535 (5)	2,341/43,575 (5)
Unfractionated heparin (%)	22,494/71,674 (31)	7,765/34,843 (22)	21,872/74,086 (30)	8,000/36,899 (22)	24,237/84,115 (29)	9,238/43,244 (21)	25,035/88,074 (28)	9,802/47,162 (21)	21,363/78,593 (27)	8,886/43,586 (20)
Glycoprotein 2b/3a inhibitor (%)	7,706/72,746 (11)	2,189/35,335 (6)	7,454/75,581 (10)	2,294/37,568 (6)	8,572/86,662 (10)	2,703/44,365 (6)	9,231/89,897 (10)	3,140/48,039 (7)	8,110/79,837 (10)	2,951/44,210 (7)
IV nitrate (%)	13,609/71,603 (19)	5,730/34,875 (16)	13,921/74,087 (19)	6,017/37,007 (16)	15,637/84,152 (19)	7,247/43,383 (17)	16,249/88,108 (18)	7,593/47,245 (16)	13,574/78,515 (17)	6,513/43,588 (15)
IV beta-blockers (%)	1,782/72,115 (2)	756/35,085 (2)	1,686/74,882 (2)	734/37,255 (2)	1,833/85,508 (2)	849/43,873 (2)	2,010/88,905 (2)	932/47,629 (2)	1,812/78,904 (2)	818/43,786 (2)
MRA (%)	4,009/53,138 (8)	1,889/25,604 (7)	4,261/54,674 (8)	1,967/26,954 (7)	4,803/62,002 (8)	2,229/31,413 (7)	4,951/64,651 (8)	2,446/33,822 (7)	4,390/56,140 (8)	2,253/30,833 (7)
Aspirin (%)	64,366/66,377 (97)	29,147/30,660 (95)	68,652/70,678 (97)	31,834/33,375 (95)	78,949/81,283 (97)	37,850/39,804 (95)	82,501/85,039 (97)	41,049/43,074 (95)	79,157/81,305 (97)	41,261/43,025 (96)
P2Y12 inhibitor (%)	71,151/80,935 (88)	33,315/39,089 (85)	75,186/85,538 (88)	35,609/42,206 (84)	86,519/99,158 (87)	42,226/50,237 (84)	89,753/103,733 (87)	45,586/54,586/54,674 (84)	83,834/98,066 (85)	44,145/53,270 (83)
Statins (%)	70,157/81,367 (86)	31,312/39,212 (80)	74,890/86,419 (87)	34,288/42,565 (81)	86,503/99,964 (87)	40,892/50,700 (81)	90,179/104,138 (87)	44,763/54,994 (81)	87,188/98,956 (88)	45,498/53,978 (84)
ACE inhibitors/ARB (%)	64,085/82,205 (78)	28,537/39,530 (72)	68,121/87,175 (78)	30,853/42,782 (72)	78,315/101,105 (77)	36,691/51,059 (72)	81,793/105,409 (78)	39,959/55,288 (72)	78,358/100,094 (78)	40,122/54,176 (74)
Beta-blockers (%)	61,932/81,940 (76)	28,174/39,408 (71)	65,786/86,884 (76)	30,329/42,651 (71)	75,611/100,720 (75)	35,946/50,878 (71)	78,994/104,970 (75)	38,540/55,188) (70)	75,172/99,642 (75)	37,981/53,782 (71)
Coronary angiogram (%)	61,401/81,976 (75)	22,584/39,367 (57)	64,285/86,755 (74)	24,304/42,552 (57)	73,967/100,546 (74)	28,846/50,744 (57)	76,674/104,423 (73)	31,103/54,727 (57)	72,070/99,461 (72)	31,307/53,788 (58)
Percutaneous coronary intervention (%)	40,831/82,378 (50)	13,410/39,745 (34)	42,740/87,396 (49)	14,624/42,997 (34)	48,969/101,325 (48)	17,351/51,414 (34)	51,952/105,585 (49)	19,140/55,600 (34)	49,103/101,238 (49)	19,582/55,028 (36)
CABG surgery (%)	2,033/66,510 (3)	471/31,166 (2)	2,332/69,952 (3)	486/33,603 (1)	2,646/80,556 (3)	637/39,975 (2)	2,878/83,601 (3)	812/43,210 (2)	2,697/77,933 (3)	770/41,871 (2)
Revascularization (CABG surgery/PCI) (%)	42,768/82,378 (52)	13,862/39,745 (35)	44,965/87,396 (51)	15,091/42,997 (35)	51,511/101,325 (51)	17,962/51,414 (35)	54,705/105,568 (52)	19,926/55,600 (36)	51,686/101,238 (51)	20,323/55,028 (37)
In-patient mortality (%)	4,867/83,821 (6)	3,811/40,442 (9)	4,845/89,021 (5)	4,021/43,858 (9)	5,779/103,282 (6)	4,747/52,385 (9)	6,004/107,645 (6)	5,307/56,791 (9)	5,522/103,016 (5)	4,698/56,159 (8)
Thirty-day mortality (%)	5,785/83,821 (7)	4,355/40,442 (11)	5,953/89,021 (7)	4,617/43,858 (11)	7,028/103,282 (7)	5,425/52,385 (10)	7,274/107,645 (7)	6,058/56,791 (11)	6,769/103,016 (7)	5,376/56,159 (10)
One-year mortality (%)	11,785/83,821 (14)	8,861/40,442 (22)	12,395/89,021 (14)	9,574/43,858 (22)	14,651/103,282 (14)	11,398/52,385 (22)	15,543/107,645 (14)	12,556/56,791 (22)	14,599/103,016 (14)	11,563/56,159 (21)
Cardiac mortality (%)	4,020/83,821 (5)	3,073/40,442 (8)	4,014/89,021 (5)	3,398/43,858 (8)	4,816/103,282 (5)	3,935/52,385 (8)	5,029/107,645 (5)	4,341/56,791 (8)	4,613/103,016 (4)	3,881/56,159 (7)
Reinfarction (%)	1,146/76,550 (1)	494/36,959 (1)	1,189/80,915 (1)	615/39,830 (2)	1,345/97,294 (1)	726/51,215 (1)	1,345/97,294 (1)	756/51,215 (1)	1,300/91,529 (1)	618/49,480 (1)
Major bleeding (%)	1,235/81,218 (2)	853/39,214 (2)	1,410/85,888 (2)	875/42,340 (2)	1,502/99,561 (2)	1,004/50,574 (2)	1,606/103,931 (2)	1,211/54,724 (2)	1,502/98,843 (2)	991/53,780 (2)
MACE (%)	5,762/83,821 (7)	4,142/40,442 (10)	5,818/89,021 (7)	4,466/43,858 (10)	6,881/103,282 (7)	5,239/52,385 (10)	7,061/107,645 (7)	5,837/56,791 (10)	6,567/103,016 (6)	5,156/56,159 (9)

*Abbreviations:* IV; intravenous, MRA; mineralocorticoid receptor antagonist, ACE: angiotensin-converting-enzyme, ARB; angiotensin receptor blockers, CABG; coronary artery bypass graft, PCI; percutaneous coronary intervention, MACE; major adverse cardiovascular events.

Unadjusted in-hospital mortality (Quintile 5; Men 5%, Women 8% vs Quintile 1; Men 6%, Women 9% (*P* < .001)), unadjusted 30-day mortality (Quintile 5; Men 7%, Women 10% vs Quintile 1; Men 7%, Women 11% (*P* < .001)), and unadjusted 1-year mortality (Quintile 5; Men 14%, Women 21% vs Quintile 1; Men 14%, Women 22% (*P* < .001)) were significantly higher in women than in men.

### Adjusted Clinical Outcomes and Adjusted Probabilities of Mortality according to Sex and Socioeconomic Status

Adjusting for the same model as before, there was no significant difference in in-hospital mortality, MACE, or 30-day mortality in women-only when comparing the most deprived Quintile with the most affluent Quintile (Table 6). One-year mortality was more likely in women from the most deprived Quintile when compared with the most affluent (Odds Ratio (OR): 1.09, 95% CI; 1.03–1.15, *P* < .001). The probability of in-hospital mortality from our adjusted model was only minimally different (Quintile 1; Men 5.8%; 95% CI 5.6–6.1%, *P* < .001, Women; 5.7%; 95% CI 5.4–5.9%, *P* < .001) (Quintile 5; Men 6.0%; 95% CI 5.8–6.3%, *P* < .001, Women; 5.9%; 95% CI 5.6–6.1%, *P* < .001) between different deprivation Quintiles and sexes (Table 7). The probability of 30-day mortality from our adjusted model was minimally higher in men in the most deprived quintile, (Quintile 5; Men 7.6%; 95% CI 7.3–7.8%, *P* < .001, Women; 7.0%; 95% CI 6.8–7.3%, *P* < .001), whereas there was no significant difference detected in more affluent Quintiles (Quintile 1; Men 7.1%; 95% CI 6.8–7.4%, *P* < .001, Women; 6.9; 95% CI 6.6%–7.1%, *P* < .001).

**Table 6.** Adjusted Outcomes for Each Quintile of Deprivation Compared to the Most Affluent Quintile (Quintile 1) for Women.

Outcome Variables	Quintile 2 (n = 199,841)		Quintile 3 (n = 199,669)		Quintile 4 (n = 199,952)		Quintile 5 (Most Deprived) n = 200,631)	
	OR	*P*	OR	*P*	OR	*P*	OR	*P*
Primary outcomes								
MACE (in-hospital)	1.04 (.95–1.15)	.402	0.98 (.90–1.07)	.671	1.08 (.98–1.18)	.137	1.07 (.96–1.18)	.208
In-hospital mortality	1.01 (.91–1.13)	.800	0.98 (.89–1.09)	.742	1.06 (.94–1.18)	.322	1.08 (.96–1.21)	.200
Thirty-day mortality	1.02 (.94–1.11)	.595	0.99 (.92–1.08)	.879	1.02 (.94–1.11)	.621	1.04 (.95–1.13)	.369
One-year mortality	1.02 (.96–1.07)	.520	1.04 (.99–1.10)	.140	1.07 (1.02–1.13)	.012	1.09 (1.03–1.15)	.004

Each quintile of deprivation is compared against our reference group ‘Quintile 1’, which is our most affluent quintile according to IMD score. Adjusted for: age, year, ethnicity, heart rate, blood pressure, history of AMI, co-morbid conditions (family history of coronary artery disease, hypertension, hypercholesterolemia, diabetes, chronic renal failure, smoking, history of asthma or chronic obstructive pulmonary disease (COPD) history of cerebrovascular accident (CVA) or peripheral arterial disease (PAD), pharmacotherapy (prescription of low molecular weight heparin (LMWH), warfarin, aspirin, ACE inhibitor, statins, beta-blockers and P2Y12 inhibitor), cardiac arrest and procedures including invasive coronary angiography (ICA) and revascularization (by percutaneous coronary intervention (PCI) or coronary artery bypass graft surgery (CABG) during admission).

**Table 7.** Margins for Adjusted in-hospital Mortality and 30-Day Mortality for Each IMD Quintile and Sex.

IMD Score Quintile and Sex Combination	Probability of in-Hospital Mortality from Adjusted Model (with 95% CI)	*P*	Probability of 30-Day Mortality from Adjusted Model (with 95% CI)	*P*	Probability of 1-Year Mortality From Adjusted Model (with 95% CI)	*P*
Quintile 1 (most affluent) and male	5.8% (5.6–6.1%)	<.001	7.1% (6.8–7.4%)	<.001	15.0% (14.8–15.6%)	<.001
Quintile 1 (most affluent) and female	5.7% (5.4–5.9%)	<.001	6.9% (6.6–7.1%)	<.001	14.5% (14.2–14.9.7%)	<.001
Quintile 2 and male	5.7% (5.4–5.9%)	<.001	7.1% (6.8–7.3%)	<.001	15.4% (15.0–15.8%)	<.001
Quintile 2 and female	5.8% (5.5–6.0%)	<.001	7.0% (6.7–7.3%)	<.001	14.7% (14.4–15.1%)	<.001
Quintile 3 and male	5.9% (5.7–6.1%)	<.001	7.3% (7.0–7.6%)	<.001	15.8% (15.4–16.1%)	<.001
Quintile 3 and female	5.7% (5.4–5.9%)	<.001	6.9% (6.6–7.1%)	<.001	14.9% (14.6–15.3%)	<.001
Quintile 4 and male	6.0% (5.7–6.1%)	<.001	7.4% (7.1–7.6%)	<.001	16.4% (16.0–16.7%)	<.001
Quintile 4 and female	5.8% (5.5–6.0%)	<.001	6.9% (6.7–7.2%)	<.001	15.2% (14.8–15.6%)	<.001
Quintile 5 (most deprived) and male	6.0% (5.8–6.3%)	<.001	7.6% (7.3–7.8%)	<.001	16.9% (16.5–17.3%)	<.001
Quintile 5 (most deprived) and female	5.9% (5.6–6.1%)	<.001	7.0% (6.8–7.3%)	<.001	15.5% (15.1–15.9%)	<.001

Probability for in-hospital, 30-day and 1-year mortality adjusted for; age, year, ethnicity, heart rate, blood pressure, history of AMI, co-morbid conditions (family history of coronary artery disease, hypertension, hypercholesterolemia, diabetes, chronic renal failure, smoking, history of asthma or chronic obstructive pulmonary disease (COPD) history of cerebrovascular accident (CVA) or peripheral arterial disease (PAD), pharmacotherapy (prescription of low molecular weight heparin (LMWH), warfarin, aspirin, ACE inhibitor, statins, beta-blockers and P2Y12 inhibitor), cardiac arrest and procedures including invasive coronary angiography (ICA) and revascularization (by percutaneous coronary intervention (PCI) or coronary artery bypass graft surgery (CABG) during admission).

The probability of 1-year mortality in our adjusted model was higher in men throughout all quintiles of deprivation (Quintile 1; Men 15.0%; 95% CI 14.8–15.6%), *P* < .001, Women; 14.5%; 95% CI 14.2–14.9%, *P* < .001) (Quintile 5; Men 16.9.7%; 95% CI 16.5–17.3%, *P* < .001, Women; 15.5%; 95% CI 15.1–15.9%, *P* < .001).

## Discussion

Our study investigating the intersection of socioeconomic disparity and sex in the management and outcomes of AMI revealed a range of important findings. Firstly, compared with the most affluent group, patients from the most deprived group received a poorer quality of care, were less likely to be treated with P2Y12 inhibitors, undergo ICA or undergo revascularization (by PCI or CABG surgery) while an in-patient, which was particularly evident in women. Secondly, after adjusting for a wide range of variables, SES was associated with higher odds of in-hospital mortality, MACE or 30-day mortality and 1-year mortality in the overall population but in women only, this relationship was not as clear, with only higher odds of 1-year mortality seen. Finally, when assessing the probability of in-hospital mortality and 30-day mortality from our adjusted model across the spectrum of SES, sex did not exert a significant effect. Furthermore, when assessing longer-term mortality, this relationship reverses, with male sex associated with higher 1-year mortality across the spectrum of socioeconomic status. Overall, women from the most socioeconomically deprived regions did not experience poorer in-hospital and longer-term outcomes than men.

Previous studies focussing on the intersection between sex and deprivation in the context of AMI have important limitations. Many of these studies have focused on the US, where it has consistently been shown how patients with lower socioeconomic status receive a poorer quality of care and have poorer outcomes following AMI,^17-19^ with these studies often only using single measures of deprivation such as household income. This same relationship exists in Canada, where Moledina et al. and Blais et al. have shown that low SES is associated with increased mortality post-AMI.^20,21^ There have been a limited number of studies into the impact of socioeconomic deprivation on AMI outcomes within the UK. Thorne et al. showed that in Wales, patients suffering AMI in the most deprived Quintile (by Welsh Index of Multiple Deprivation score (WIMD)) had higher 30-day mortality when compared with patients in the most affluent Quintile,^9^ however, within studies from the UK particularly, there is a lack of analysis of the longer-term outcomes post-AMI according to level of deprivation.

The impact of sex on the processes of care and mortality post-AMI is an area with discordant results. Wilkinson et al. have demonstrated that women in the UK post-AMI have poorer rates of prescription of guideline-directed medical therapy (GDMT) and significantly higher mortality.^22^ This contrasts with a nationwide study of post-AMI patients in Sweden, which suggested that after adjusting for comorbidities, there was no significant difference in ‘short-term outcomes’ between women and men.^23^ In Europe, there is more consistency in the longer-term outcomes of AMI according to sex. An analysis of 5-year mortality post-AMI in Norway suggests lower adjusted all-cause mortality in women compared with men with non-ST segment myocardial infarction (NSTEMI)^24^ and in German AMI patients, a small increase in unadjusted in-hospital mortality in women was seen, which diminished after adjustment.^25^ A meta-analysis of all eligible studies of sex-based differences on the longer-term AMI outcomes suggested that the differences seen in longer-term mortality are predominantly explained by age, comorbidity, and treatment utilization between the sexes.^26^

There is growing appreciation of the importance of the social determinants of health (SDOH), referring to the impact on your health of the environment into which you are born and live in, with a clear, prevailing relationship between lower socioeconomic position and poorer health.^27^ A Scientific Statement from the American Heart Association (AHA) considers six key domains for the SDOH; socioeconomic position (SEP), race and ethnicity, social support, culture, access to medical care and residential environment,^28^ with SEP encompassing multiple elements such as education, income, and employment. ‘Greater social adversity’, used interchangeably with adverse SDOH has been linked to both a higher burden of cardiovascular risk factors, and poorer cardiovascular outcomes.^29^ The relationship between socioeconomic deprivation and coronary artery disease (CAD) outcomes has predominantly been investigated in the US, where studies have repeatedly shown that patients from more socioeconomically deprived areas are less likely to receive guideline recommended treatments and have poorer in-hospital and longer-term outcomes post-AMI and with stable CAD.^18,30,31^

In North American Healthcare systems, such as the US, women have more financial barriers to medical care according to cost and insurance status,^32,33^ which may be a major contributor to the documented disparities in access to invasive therapies and poorer clinic outcomes in women in the US.^2^ Furthermore, within the US, we have shown that women are more likely to be within the lowest median household income group,^19^ which will be a further contributor to these sex-based disparities. However, this does not appear to just be a US-based phenomena, even in affluent European insurance-based systems such as Italy, there appears to be poorer short-term outcomes post-acute coronary events in women compared with men in the most deprived regions.^10^ Our study highlights that in the universal healthcare system of the UK, SES does not affect access to high-quality post-AMI care and clinical outcomes such as in-hospital and 30-day mortality more in women than in men. Prior studies have demonstrated the role of universal healthcare in eliminating disparities in AMI outcomes according to SES, Danchin et al. showed how the implementation of free, comprehensive medical coverage for low-income patients in the early 2000s in France, eliminated the inequalities in acute and ‘mid-term’ AMI outcomes between patients of differing socioeconomic status.^34^ Introducing universal healthcare for AMI patients was also shown to increase prescription of GDMT and use of PCI in the Chilean healthcare system, significantly reducing health inequalities,^35^ so we suggest that the universal healthcare system in the UK is a major contributor to this. We also acknowledge that improved education and awareness of the atypical way in which AMI can present in women will be mitigating previously noted sex-based disparities in the recognition and treatment of AMI. Further work needs to be undertaken to understand why the most socioeconomically deprived patients still experience poorer processes of care and outcomes post-AMI in the UK, particularly regarding longer-term outcomes in men in areas of socioeconomic deprivation.

## Strengths

Myocardial Ischaemia National Audit Project is a comprehensive registry, capturing every admission with AMI in the UK between 2008 and 2019. The UK has a publicly funded, universal healthcare system, where regional disparities in access to treatments should be minimized in contrast to insurance-based systems such as in the US,^5^ therefore, the results should be generalizable to the entire UK population. Most recent UK studies using MINAP have not had longer-term mortality data available, and the addition of ONS mortality data to such a comprehensive registry is a strength to our study.

## Limitations

There are important limitations to observational studies of this type. The MINAP data registry shares the weakness of other national registries, including the self-reporting of adverse events with no external validation. Although the MINAP dataset included important clinical and demographic variables of interest, there are limitations to the data collected. For example, the database does not capture frailty score, severity of CAD, psychosocial risk factors, access to the use of healthcare, rationale for specific medications, or an exhaustive comorbidity list. Furthermore, the database does not capture markers of inflammation, biomarkers, low density lipoprotein (LDL) cholesterol levels, or less common risk factors such as malignancy, lipoprotein(a), or clonal haematopoiesis of indeterminate potential. Furthermore, inherent to large registries such as MINAP is the issue of missing data, which we have attempted to mitigate by using MICE. Our ONS mortality data is comprehensive for patients who died in England, there may be a small number of patients that have moved to alternate countries within the UK or elsewhere that we are unable to capture, The MINAP registry has a record of the IMD 2010 score for each patient but does not record components of the score, therefore, we are unable to ascertain which components of the score are having the most significant impact on outcomes. MINAP included comprehensive data from the IMD 2010 score, we acknowledge that ideally, we would have used a more recent IMD score but note there is minimal temporal change demonstrated in the IMD score.^6^ We acknowledge that AMI is a heterogenous group of patients, and we could be missing important intersections between gender and socioeconomic deprivation in ST-segment myocardial infarction (STEMI) or NSTEMI patients, this is important as recent trials have suggested that although the adjusted prognosis post-AMI has been better in women, in STEMI, particularly in younger women, there appears to be a poorer prognosis when compared with men.^36^

## Conclusion

Our study demonstrated that women were significantly older, less likely to be from an ethnic minority background and were less likely to undergo ICA or PCI for all levels of socioeconomic deprivation. Patients from the most socioeconomically deprived Quintile had higher in-hospital, 30-day and 1-year mortality compared with the most affluent, but this relationship was weaker in women. Male sex predicted poorer longer-term outcomes post-AMI. Overall, our study has shown that sex did not significantly influence the effect of deprivation on AMI processes of care and outcomes in a universal healthcare system.

## Data Availability

The National Institute for Cardiovascular Outcomes Research (NICOR) provided the data underlying this article. Data will be shared on request to the corresponding author with the permission of NICOR.
